# Prevalence and predictors of child labour among junior public secondary school students in Enugu, Nigeria: a cross-sectional study

**DOI:** 10.1186/s12889-021-11429-w

**Published:** 2021-07-07

**Authors:** N. O. Enebe, J. T. Enebe, C. C. Agunwa, E. N. Ossai, U. E. Ezeoke, C. A. Idoko, C. O. Mbachu

**Affiliations:** 1 Department of Community Medicine, University of Nigeria Teaching Hospital (UNTH), Enugu, Nigeria; 2 Department of Obstetrics and Gynaecology, Enugu State University of Science and Technology, College of Medicine / Teaching Hospital, P.M.B. 1030, Parklane, Enugu, 400001 Nigeria; 3 Department of Community Medicine, College of Health Sciences, Ebonyi State University Abakaliki, Abakaliki, Nigeria

**Keywords:** Child labour, Secondary school, Enugu, Metropolis, Nigeria

## Abstract

**Background:**

Globally, millions of children are involved in child labour. However, low- and middle-income countries are mostly hit. This study examined the predictors of child labour among public secondary school students in the Enugu metropolis.

**Methods:**

This was a descriptive cross-sectional study of 332 junior secondary students attending public schools in Enugu metropolis, Nigeria. Multistage sampling technique was used to select the six secondary schools and the students that participated in the study. Data collection was done from September to October 2018. Pretested structured, interviewer-administered questionnaire was used for data collection. The questionnaire contained information on the sociodemographic variables, the kind of work done by the respondents and the number of working hours spent weekly. UNICEF’s standard indicator for child labour was used to estimate the prevalence of child labour. Logistic regression was used to identify socioeconomic predictors of child labour.

**Results:**

The prevalence of overall child labour was 71.7%, while for domestic and economic child labour prevalence were 52.1 and 34.0%, respectively. About 35.2% of the respondents worked under hazardous conditions while 8% were forced to work. Two-thirds (236, 65%) of the respondents who have heard about child labour perceived it as wrong. The child labourers mainly worked to render financial assistance to their parents. The predictors of child labour were class of study (AOR = 2.208 (95% CI: 1.199–4.066) and weekly income earned (AOR = 0.316 (95% CI: 0.176–0.567).

**Conclusion:**

The prevalence of child labour among junior students in public secondary schools in Enugu is high, and is predicted by the level of schooling and income earned. Economic and social reforms could contribute to addressing the predictors of child labour.

**Supplementary Information:**

The online version contains supplementary material available at 10.1186/s12889-021-11429-w.

## Background

The United Nations Conventions on the rights of the child (CRC) defines a child as a person below the age of eighteen years [1, 2]. The Convention emphasizes the need for children to be protected from violence, sexual exploitation, and abuse as well as from work exploitation and hazardous jobs [1, 3]. United Nations Children’s Fund (UNICEF) defined child labour as work that is physically, mentally, socially or morally dangerous and harmful to children and tends to deprive them of opportunities for development and schooling [4]. Moreover, the International Labour Organization (ILO) defines child labour as work that deprives the child of his childhood potential and dignity or is harmful to his physical and mental development [3]. It is a well-known fact that child labour impacts negatively on the education of children, often causing them to drop out of school or spend more years in school. Children who engage in child labour are more likely to miss out on school days and lag behind their peers in academic performance. Hence, child labour is an infringement on the right of a child to quality education [5].

It is important to note that not all types of work done by children are regarded as child labour. This is particularly imperative in low and middle-income countries such as Nigeria where it is a tradition for children to work alongside their parents or guardians [6]. In 2008, the International Conference of Labour Statisticians (ICLS) adopted an operational definition of child labour that includes measurement of working time, age-specific thresholds and nature of work performed [7]. UNICEF and ILO also developed standard indicators for child labour disaggregated into three age categories, and these include, (i) for ages 5–11 - at least one hour of economic work or 28 h of domestic work per week; (ii) for ages 12–14 - at least 14 h of economic work or 28 h of domestic work per week; and (iii) for ages 15–17 - at least 43 h of economic or domestic work per week [8]. The Nigerian Multiple Indicator Cluster survey (NMICS) applied this module for the assessment of child labour in the year 2016/2017 [9].

Child labour remains a major public health issue in Nigerian despite prohibitions in law [10]. It has been estimated that about 15 million of children under the age of 14 years in Nigeria are engaged in one form of work [11]. The levels of practice of child labour in Nigeria has been high and varies across zones [12, 13]. The Northern region is said to be child labour endemic due to the “misapplication of the Almajiri system”. Consequently, the northern region is most affected by out-of-school children [5]. In the south-eastern and south-south geopolitical zones, many children drop out of school to work as domestic help. In the western zone, many children are involved in street hawking and as domestic help [13]. Further, some Nigerian children are engaged in an occupation such as mining, quarrying, and agriculture [14].

In Nigeria, child labour is driven by social, demographic and economic factors such as poverty and loss of employment of parents, rural-urban migration, large family size and cultural norms such as polygamy [15]. Other drivers include maldistribution of schools, poor accessibility, and high cost of tuition [15]. More recently, conflicts and terrorism have caused internal displacements of people and damage to school facilities, pushing more children into child labour. Moreover, the mass killings of communities by bandits in northern Nigeria have contributed to creating more orphans and potential victims of child labour [16, 17].

Studies done in different parts of Nigeria show that child labourers experience problems such as school drop-out, sexual molestation, and robbery [18–20]. The vulnerability of child labourers to poor education and health outcomes threaten the achievement of Sustainable Development Goals (SDGs) [21]. Considerable proportions of school-aged children in Nigeria engage in child labour while they are in school, and the majority of child labourers are in school [15]. Therefore, child labour impacts school attendance and performance for in-school children.[18,19, 22].

In 2011, the overall National prevalence of child labor was 47% among children aged 5–14 years and 76% among children who were engaged in child labor but were attending school. Enugu state with an overall child labour prevalence of 47.9% with 86% of them combining child labour with school, was found to be among the 20 states with prevalence greater than the national average [15]. From the information above, it can be deduced that being in school does not rule out the possibility of being a child labourer; majority of child labourers are in-school with the resultant effect of poor school attendance and performance [18, 19, 22].

Enugu State introduced Universal Basic Education (UBE) into her education system in the year 1999 and despite that the number of out-of-school children aged 3-18 years was 26.5%, 15 years after that initiation of the programme [23]. The major reason for this large number of school children being out of school was financial in most cases [23] and many of those in school likely engage in so many economic activities to help them alleviate their financial needs while schooling. Inclusive Education and other programmes were also introduced in Enugu state in 2014 and beyond [24, 25] to help drive all children to school and also to discourage child labour among school children. These efforts are meant to have reduced the number of out-school-children in the state and also the number of children that engage in child labour while schooling hence the need for this study.

ILO [26] noticed that much studies have evaluated the impact of child labour and school enrollment while less is known about the relationship between child labour and school attendance. This is because it is easier to elicit school enrollment from household survey than school attendance. This gives confidence that students school environment can be used to study the different forms of child labour and their prevalence in our environment hence this study is rather school-based.

Enugu therefore presents a perfect setting to study the current prevalence of child labour and issues promoting this public health menace in Nigeria. This study will also bring to view the effect of government interventions in the past towards reducing the number of children that are combining schooling with child labouring or dropping out of school due to child labour.

The current study estimates the prevalence of child labour in public secondary schools and underscores the predictors of child labour in this population. This will contribute to existing evidence on the burden of child labour. Furthermore, uncovering the predictors of child labour among schooling children could inform the formulation of policies and design of intervention strategies to reduce child labour.

## Methods

### Study design

This was a descriptive cross-sectional study of 332 junior secondary schools (JSS) students in public secondary schools in the Enugu metropolis, using the quantitative research method.

### Study setting

This study was conducted in the metropolis of Enugu state. The State is one of the five states in south-eastern Nigeria. There are 17 Local Government Areas (LGAs) in the State, and three of them make up the metropolis. There are 314 public secondary schools and 1382 private secondary schools in Enugu State. The student-teacher ratio and student-class room ratio in the public secondary schools are 16:1 and 232:1, respectively. Enugu metropolis harbours the majority of the economic activities in the State. The metropolis also has the highest numbers of primary, secondary and tertiary institutions in the State.

Recruitment of participants and data collection was done from September to October 2018.

### Study population

The study population consisted of junior secondary students in public secondary schools. Participants were selected from the junior classes to reflect only the population that was being covered by the free education of UBE. Furthermore, JSS 2 and 3 classes were purposively chosen because the researchers considered that these students would be mature enough to partake in some meaningful interaction with researchers, and would be bold enough to provide answers to the questions. Senior secondary students were excluded because they were not covered by the free education policy of UBE, and they were more likely to be above the upper age limit (17 years) for the child labour questionnaire [18, 19, 27].

#### Exclusion criteria

The following groups of students were excluded from participating in the study: students who were too ill to respond to questions (acutely ill); students with known chronic diseases for which they regularly missed school to go for medical check-ups; students who were absent from school on the day of the survey; and newly enrolled students who were not in school in the previous term.

##### Sample size calculation

The minimum sample size for the study was determined using the formula for estimating sample size for descriptive studies *N* = Z2pq/d2 [28]. From a previous study in Ogun state, 68.6% of the school children practised child labour [27]. A total of 332 junior secondary school students were studied based on a type 1 error (α) of 0.05 in a two-sided test with a power of 0.8.

##### Sampling technique

This study used a multistage sampling technique. Enugu metropolis is made up of 3 LGAs. A list of all the public secondary schools in each of the three LGAs was obtained and this formed the sampling frame for the study. At the first stage, two public secondary schools were randomly selected from each of the three LGAs in the Enugu metropolis and this gave a total of 6 schools that were used for the study. The six schools were found to comprise 2 boys’ schools, 2 girls’ schools and 2 co-educational schools. At the second stage, an equal number of students (62 students per school) were selected from each of the 6 selected schools given a total of 372 students that were eligible and selected for the study. Also, an equal number of students were selected from each of JSS 2 and 3 classes (31 students from each class). Within each class, a list of all eligible students was obtained and a systematic sampling technique was employed to select 31 students from each class of JSS 2 and JSS3. Where there was more than one arm/stream in a class, simple random sampling was done to obtain one arm/stream from that class. In a situation where less than 31 students met the criteria in a selected class, participants were selected from all arms using proportional allocation.

##### Study instruments

A pre-tested, structured, interviewer-administered questionnaire was used to collect data from the eligible students. The questionnaire was adopted from the concise revised standard Multiple Indicator Cluster Sampling (MICS) module on child labour [29]. The questionnaire elicited socio-demographic characteristics of participants, as well as the kind of work they did and the number of hours they worked per week. See additional file 1.

#### Data collection and measurement of variables

##### Pre-testing of the questionnaire

The structured self-administered questionnaire was pre-tested among 45 pupils (10% of the sample size) in a public secondary school within the metropolis, that was not selected for the study. The pre-testing was used to ensure that the questions were clear, complete and appropriately framed/structured. The questionnaire was revised accordingly at the end of the pre-testing.

The prevalence of child labour was obtained by adopting the UNICEF’s standard indicator for child labour [9] which uses the MICS module on child labour [29] These tools assess child labour as the percentage of children aged 5–17 years with a total work hour above the given age-specific threshold in the previous week before the study. Those defined as child labourers were, therefore, (i) children aged 5–11 who performed at least one hour of economic work or 28 h of domestic work per week, (ii) children aged 12–14 who performed at least 14 h of economic work or 28 h of domestic work per week, and (iii) children aged 15–17 who performed at least 43 h of economic or domestic work per week [8].

##### Perception of child labour

This was measured by asking respondents if they had heard about child labour, what constituted child labour, and whether child labour was right or wrong.

##### Measurement of variables

The dependent variable/outcome variable was the presence or absence of child labour. The independent/predictor variables included respondents’ socio-economic status (SES), age, sex, class, family structure, position in the family, household size, type of work performed by a child, number of working hours, and weekly income generated. SES was determined by the “Wealth Index Scale” which used information on the ownership of some vital household properties (such as television, refrigerator, and car.) to reflect the standard of living of the household [30].

#### Data analysis

Data entry and analysis were done using IBM Statistical Package for Social Sciences (SPSS) version 23 and STATA software. The STATA software was used only for the calculation of the wealth index (socioeconomic status) of the participant’s families while other remaining analyses were done with SPSS software. Univariate and bivariate analyses were performed. The association between the independent variable and categorical dependent variables was tested using Chi-square. Associations that returned a *p*-value of 0.05 or less were entered into the logistic regression model to determine predictors of child labour.

The logistic regression analysis results were reported using the adjusted odds ratio (AOR), 95% confidence interval, and the level of statistical significance was set at a *p*-value of < 5%. All monetary calculations involving the use of ‘The Naira’ was done based on a conversion rate of ₦370 equal to one United States Dollar (USD) as obtained at the time of the study.

The STATA statistical software version 12 was conveniently used to generate the socio-economic status index using Principal Component Analysis, (PCA). This involved inputting variables related to ownership of ten household items that included radio, television, air conditioner, car, fridge, generator, electric fan, phone, rechargeable light, and electric iron. Quartiles, (Q) was used for the calculation of distribution cut off points with each respondent assigned the wealth index score of the household. The quartiles were the poorest (Q1), the very poor (Q2), the poor (Q3), and the least poor(Q4). These were further sub-divided into low socioeconomic class (the poorest and very poor) and high socio-economic class (the poor and the least poor) groups [31].

The primary outcome measure of the study was the prevalence of child labour among the respondents who were attending JSS2 and JSS3 in public secondary schools in Enugu metropolis, Nigeria.

## Results

### Socio-demographic characteristics of respondents

Out of 372 eligible students, parents of 18 participants (4.8%) did not give consent for the study while 22 (5.9%) participants did not complete their questionnaires properly. It was a total of 332 students (89.2%) who completely filled their questionnaires that was used for analysis. The socio-demographic characteristics of the respondents as shown in Table 1 revealed that the mean age of the participants was 14 ± 1.2 years with the majority (73.8%) falling within the age range of 12 to 14 years. There were more males than females (53.6% vs 46.4%). All respondents were Christians and the majority (98%) were from the Igbo ethnic group. The majority (92.5, 89.2%) were from the monogamous household type and nuclear family structure respectively. A large proportion (78%) lived with at least one parent, 63.3% lived with both parents while 28% lived with either other relatives or unrelated custodians. The respondents mainly had a large household size of 5 to 9 (79.5%) while the majority (75.3%) fell within 1 to 4 birth order.

**Table 1:** Socio-demographic characteristics of respondents

Variables	category	Frequency (***n*** = 332)	Percent
**Sex**	Male	178	53.6
	Female	154	46.4
**Ethnicity**	Igbo	325	97.9
	Minority eg. Ijaw	7	2.1
**Age category (years)**	9–11	9	2.7
	12–14	245	73.8
	15–17	78	23.5
	Mean age (SD) = 14 (1.2)		
**Household size category**	1–4	37	11.1
	5–9	264	79.5
	10+	31	9.3
**Total number of children category**	1–4	101	30.4
	5+	231	69.6
**Birth order category**	1–4	250	75.3
	5+	82	24.7
**Custodian of the respondents**	Both parents	210	63.3
	Single mother	30	9.0
	Single father	3	.9
	Other relatives	55	16.6
	Unrelated guardian	34	10.2
**Employment status of the father**	Petty trading	50	15.1
	Big business	58	17.5
	Public servants	61	18.4
	Employed in a private firm	17	5.1
	Artisan / Self-employed	110	33.1
	Farmer	20	6.0
	Unemployed	16	4.8
**Employment status of the mother**	Petty trading	101	30.4
	Big business	50	15.1
	Public servants	55	16.6
	Employed in a private firm	2	8.1
	Artisan / Self-employed	59	17.8
	Farmer	25	7.5
	Unemployed	15	4.5
**Employment status of the male custodian**	Petty trading	25	26.9
	Big business	24	25.8
	Public servants	12	12.9
	Employed in a private firm	28	30.1
	Artisan / Self employed	1	1.1
	Farmer	2	2.2
	Unemployed	1	1.1
**Employment status of the female custodian**	Petty trading	9	9.7
	Big business	21	22.6
	Public servants	24	25.8
	Employed in a private firm	13	14.0
	Artisan / Self employed	23	24.7
	Farmer	2	2.2
	Unemployed	1	1.1

Other background characteristics shown in Table 2 revealed that the respondents’ biological parents mainly had secondary education as their highest level of education while for those not living with their parents, tertiary education was the highest level of education of their custodians. The parents of the respondents were mainly artisans, unemployed and petty traders while the custodians were businessmen, employed in private firms and public servants.

**Table 2:** Educational levels of parents/guardians

Educational levels	Frequency (***n*** = 332)	Percent
**Father**		
No formal education	6	1.8
Primary	59	17.8
Secondary	173	52.1
Tertiary	94	28.3
**Mother**		
No formal education	8	2.4
Primary	46	13.9
Secondary	178	53.6
Tertiary	100	30.1
**Male custodian**		
No formal education	0	0
Primary	9	9.7
Secondary	31	33.3
Tertiary	53	57.0
**Female custodian**		
No formal education	0	0
Primary	10	10.8
Secondary	33	35.5
Tertiary	50	53.8

### Prevalence of child labour according to the type of activities done

**Fig. 1:**
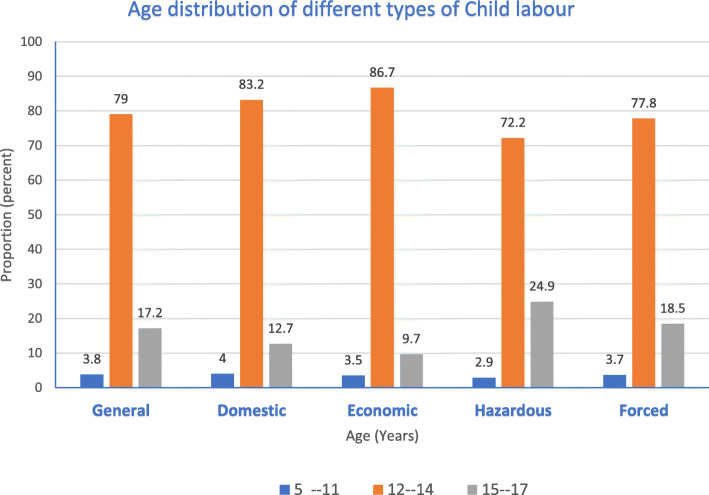
Age distribution of different categories of child labour

The prevalence of general child labour among students of public secondary schools in Enugu metropolis, Nigeria was 71.7% while the prevalence of Domestic and Economic child labour was 52.1 and 34.0% respectively. About 35.2% of the respondents worked under hazardous conditions while 8.1% were forced to work. Table 1 shows that majority of the child labourers (73.8%) fell within the age bracket 12 to14 years while only 2.7% of them fell within 9 to 11 years. Figure 1 strengthens this finding as respondents within the same age bracket (12 to 14 yrs) carried out all categories of child labour more than those that fell within 5 to 11 years and 15 to 17 years age brackets.

Table 3 shows the prevalence of child labour according to the different characteristics of the participants. The majority of the child labourers started working between the age of 5–10 years and the mean number of hours the participants worked in the preceding week before the study was 45 h. The total number of both economic and domestic work hours in the previous one week was compared in Table 4.

**Table 3:** Prevalence of child labour by different categories

Variables	Category	Child labour		Total
		**Yes (*N*** **= 332)**	**No (*N*** **= 332)**	
**Age category (years)**	5–11	10 (3.0)	0 (0)	10 (3.0)
	12–14	187 (56.7)	57 (17.2)	244 (73.5)
	15–17	41 (12.3)	37 (11.1)	78 (23.5)
**Class**	JSS2	92 (27.7)	20 (6.0)	112 (33.7)
	JSS3	146 (44.0)	74 (22.3)	220 (66.3)
**Wealth Index quartile**	Poorest	61 (18.4)	22 (6.6)	83 (25.0)
	Very poor	60 (18.1)	29 (8.7)	89 (26.8)
	The poor	58 (17.5)	22 (6.6)	80 (24.1)
	Least poor	59 (17.8)	21 (6.3)	80 (24.1)
**Age when started work (years)**	< 5	23 (6.9)	8 (2.4)	31 (9.3)
	5–10	120 (36.1)	39 (11.7)	159 (47.9)
	> 11	95 (28.6)	47 (14.2)	142 (42.8)
**Total number of children**	1–4	72 (21.7)	29 (8.7)	101 (30.4)
	> 5	166 (50.0)	65 (19.6)	231 (69.6)
**Birth order**	1–4	184 (55.4)	66 (19.9)	250 (75.3)
	> 5	54 (16.3)	28 (8.4)	82 (24.7)
**Total no of working children**	1–4	170 (51.2)	75 (22.6)	245 (73.8)
	> 5	68 (20.5)	19 (5.7)	87 (26.2)
**Household family size**	1–4	26 (7.8)	11 (3.3)	37 (11.1)
	5–9	186 (56.0)	78 (23.5)	264 (79.5)
	> 9	26 (7.8)	5 (1.5)	31 (9.3)
**Satisfied with work**	Yes	188 (56.6)	76 (22.9)	264 (79.5)
	No	50 (15.1)	18 (5.4)	68 (20.5)
**Self-ownership of business**	Yes	21 (6.3)	5 (1.5)	26 (7.8)
	No	217 (65.4)	89 (26.8)	306 (92.2)
**Custodian**	Parents	146 (44)	59 (17.8)	205 (61.7)
	Single mother	24 (7.2)	7 (2.1)	31 (9.3)
	Single father	2 (0.6)	1 (0.317)	3 (0.9)
	Other relations	41 (12.3)	17 (5.1)	58 (17.5)
	Unrelated guardian	25 (7.5)	10 (3.0)	35 (10.5)

**Table 4:** Some of the working characteristics of the respondents

Variables	Category	Frequency		percent	
Age when started work category (years)	< 5	31		9.3	
	5–10	159		47.9	
	11+	142		42.8	
	Mean (SD) = 9.73 (2.8)				
Total work hours in the previous 1wk (hours)	< 14	24		7.2	
	15–28	62		18.7	
	29–43	77		23.2	
	> 43	169		50.9	
	Median (Range) = 45 (4–145)				
**Variable**	**Category**	**Economic activities**		**Domestic activities**	
		**Frequency**	**percent**	**Frequency**	**percent**
work hours in the previous 1wk (hours)	< 1	62	18.7	0	0
	01--14	128	38.6	60	18.1
	15–28	82	24.7	109	32.8
	29–43	41	12.3	95	28.6
	> 43	19	5.7	68	20.5
	Median (Range)	28 (2–127)		12 (0–84)	

### The pattern of child labour in the previous week before the study

**Fig. 2:**
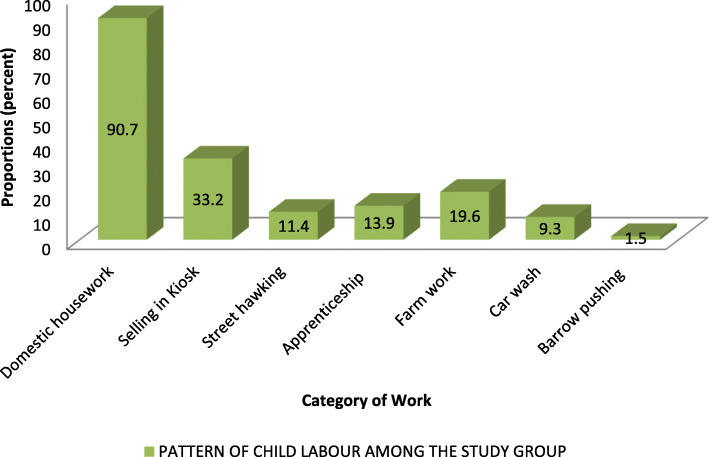
Pattern of child labour in the previous week before the study. MSK – Musculo-skeletal system

Figure 2 shows that 301 (90.7%) of all respondents carried out domestic activities in the previous 1 week before the survey, close to 20% of them spent greater than 43 h on domestic work during the same week. Other activities carried out by the respondents were selling in kiosks (33.2%), farm work (19.6%), apprenticeship (13.9%), street hawking (11.4%), car wash (9.3%) and barrow pushing at the market (1.6%).

### Perceptions of child labour among respondents

About 29% of the respondents had not heard of child labour before the study and therefore had no view about it. However, out of the 236 respondents that had heard about it, 64.8% (153) of them perceived it as wrong.

### Factors associated with child labour (overall)

Table 5 shows that class of the respondents and weekly income generated by the respondents were the predictors of child labour; Lower junior secondary class (JSS2) (AOR = 2.180 (95% CI: 1.183–4.016), *P* = 0.003) and weekly income less than 10,000 Naira (USD27) (AOR = 0.315 (95% CI: 0.175–0.565), P = < 0.001) were the predictors of child labour. Respondents who were in JSS2 were 2.2 times more likely to be involved in child labour than those in JSS3. Also, respondents who earned less than 10, 000 Naira weekly were 3.2 times less likely to be involved in child labour than those who earned more than 10,000 nairas (USD 27) weekly. Age was a factor from chi-square test (*p* < 0.001) but did not predict child labour (AOR: 1.20; CI: (0.7–2.0)).

**Table 5:** Factors associated with child labour (overall)

Variables		Overall child labour (***n*** = 332)		P-value	AOR (CI)
		Yes N (%)	No N (%)		
**Age category**	5–11	9 (100)	0 (0)	**< 0.001**	1.20 (0.7–2.0)
	12–14	188 (76.7)	57 (23.3)		
	15–17	41 (52.6)	37 (47.4)		
**Sex**	Male	134 (75.3)	44 (24.7)	0.143	1.23 (0.7–2.0)
	Female	104 (67.5)	50 (32.5)		
**Class**	JSS 2	92 (82.1)	20 (17.9)	**0.003**	**2.18 (1.2–4.0)**
	JSS3	146 (66.4)	74 (33.6)		
**Socio-Economic Class (SEC)**	Low SEC	117 (73.1)	43 (26.9)	0.626	N/A
	High SEC	121 (70.3)	51 (29.7)		
**Custodians**	Both parents	146 (71.2)	59 (28.8)	0.807	N/A
	Single parents	26 (76.5)	8 (23.5)		
	Others	66 (71.0)	87 (89.0)		
**Birth order**	1–4	184 (73.6)	66 (26.4)	0.204	N/A
	5+	54 (65.9)	28 (34.1)		
**Family size**	< 5	47 (70.1)	20 (29.9)	0.763	N/A
	5 and above	191 (72.1)	74 (27.9)		
**Number of working children**	1–4	170 (69.4)	75 (30.6)	0.204	N/A
	5+	68 (78.2)	19 (21.8)		
**Age when started work**	< 5	23 (74.2)	8 (25.8)	0.224	N/A
	5–10	120 (75.5)	39 (24.5)		
	> 10	95 (66.9)	47 (33.1)		
**Weekly Income of student**	< 1000	139 (64.7)	76 (35.3)	**< 0.001**	**0.32 (0.2–0.6**)
	1000 and above	99 (84.6)	18 (15.4)		
**Household type**	**Monogamous**	222 (72.3)	85 (27.7)	0.365	N/A
	**Polygamous**	16 (64.0)	9 (36.0)		
**Family structure**	**Nuclear**	209 (70.6)	87 (29.4)	0.211	N/A
	**Extended**	29 (80.6)	7 (19.4)		

### Challenges of child labour

**Fig. 3:**
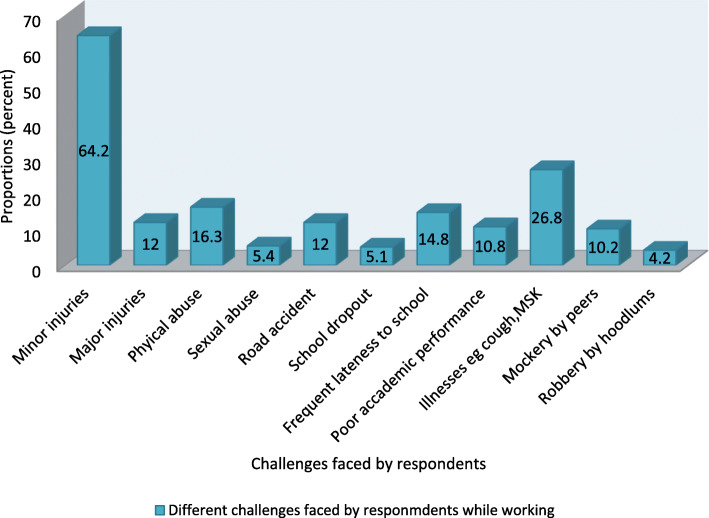
Challenges of child labour of the study participants

Figure 3 shows that the majority (64.2%) of the respondents sustained minor injuries while working, some (26.8%) of them experienced minor illnesses such as cough and musculoskeletal pains, 16.3% were physically abused, 14.8% were frequently late to school, 12% sustained major injuries and 12% were involved in road accidents, 10.8% performed poorly in their academics, 5.4% were sexually abused, 5.1% dropped out of school at some point while 4.2% were robbed.

## Discussion

The high prevalence of child labour found in this study suggests that child labour is quite common among school children in Enugu. This is similar to what has been reported by other studies among junior secondary school students in southwest Nigeria [19] [27] However, it is almost twice as much as what was reported for the whole State following the 2016/2017 MICS/NICS [9]. The difference between these findings and that of the current study could be due to the difference in methodology and data collection. While MICS/NICS is a household survey where parents were interviewed, this current study interviewed the children themselves. This is likely to reduce recall bias as well as eliminate the possibility of parental interference and influence. When compared with the National Child Labour Survey, [9] done in 2018, the prevalence obtained in the current study is also higher than what was reported among in-school children.

Assessment of the different categories of child labour among the respondents revealed that though 90.7% of the respondents carried out domestic works, only 52.1% of them carried it out above the given age-specific threshold while 34.0% of the respondents carried out economic work above the age-specific threshold. Also, 35.2% of respondents worked under hazardous conditions while 8.1% of them were forced to work. The higher prevalence of domestic works compared to economic work could be explained by the common practice of engaging underage children as domestic help in the State, and indeed the country. These children are usually brought from poor households in rural areas and neighbouring states to the urban areas to assist in caring for children, cleaning the house, and cooking. The prevalence of children engaged in economic and domestic activities obtained in this study are comparable to similar studies undertaken in southwest Nigeria [18, 19, 27] However, the variations could be explained by the differences in the overall economic viability of the study sites. Thus, studies that were done in areas with greater economic viability yielded a higher prevalence of child involvement in economic work in comparison to our study area.

Our finding that over three-quarters of the child labourers fell within the age range of 12 and 14 years is in keeping with other Nigerian studies [27, 32]. Also, the fact that 61.3% of the child labourers from this study lived with both parents, disapproves of the general view that it is mainly related and unrelated guardians that subject their wards to child labour. From the current study, child labourers living with their parent(s) were mainly involved with economic activities while those living with related and unrelated guardians were mainly involved with domestic activities.

Awareness of child labour among the respondents was below expectation, and barely two-thirds of the children perceived it as wrong. A considerable number of children perceived it as right, and this could explain the finding that many of them expressed satisfaction with the work they did and thought that child labour should be encouraged. This is in keeping with findings from other authors that reported that 54–86% of working children and their parents felt that children should work and that engaging in economic activities has no negative impacts on children [32, 33]. However, some studies have also reported inconsistent findings where over 60% of parents reported that child labour is hazardous to children, and exposes them to social vices and numerous health risks [34, 35].

The study revealed an association between child labour and age, tribe, gender, socio-economic status, custodians, family size, number of working children, weekly income of students, as well as family structure, however, only the age of the students (*p* < 0.001), class (*p* = 0.003), and weekly income made by the respondents (*p* < 0.001) were found to be significantly associated with overall child labour. Class: AOR = 2.180 (95% CI:1.183–4.016) and weekly income: AOR = 0.315 (95% CI: 0.175–0.565) were the predictors of overall child labour. Children in JSS 2 were twice more likely to practice child labour than those in JSS3. This could be explained by the fact that JSS3 is an exam class where secondary school students write their lower secondary school external exams (junior Secondary School Certificate Exam). With the high literacy level of the state, caregivers would prefer to engage children when they are not in exam classes than when they are preparing for external exams. Similarly, schools engage students in exam classes in such a way that they return home after work/business hours [36].

Child labour was also predicted by weekly income made by the child labourers as those who earned over ₦1000 (US$2.7) a day were about 3 times more likely to carry out child labour than those who earned less. This is consistent with the finding that the majority of the economic child labourers in the study worked to support themselves or their parents financially. This is also supported by the findings that about 90% of the respondents have a large family size (greater than 5) with close to 70% of them having to train more than 5 children. In addition, more than 50% of the households fell within the poorest and very poor wealth quartiles. Other authors have also reported that low socioeconomic status, poor family background, and large family size are associated with child labour [20, 27, 37]. Furthermore, child labour has been reported to increase with decreasing level of parental education and socioeconomic status [27].

This study is limited by the fact that the psychological state of the children may have affected their responses and this was not determined in the research. However, this study has given an overview of the pattern of child labour in the Enugu metropolis. A larger study that can compare urban and rural areas of the state may give a clearer picture. Also, a qualitative study will help get a detailed understanding of the experiences of these child labourers both in their workplaces and in the hands of their caregivers.

## Conclusion

The prevalence of child labour is high among junior secondary school children in public schools in the Enugu metropolis. The majority of these child labourers are engaged as domestic workers, and a significant number of them spend over 43 h a week working at home and performing tasks that are considered hazardous for their age. The predictors of child labour were the level of study and weekly income earned from the economic activities.

This study should be replicated in rural areas and other states in Nigeria to help compare the findings. We recommended further studies on the relationship between child labour and the academic performance of students. Policy draft, review, and implementation of the existing ones should be made to help address some of the issues noted in this study. Awareness creation on the consequences of child labour is urgently needed.

The establishment and sustainability of education programs such as the Universal Basic Education (UBE) scheme will reduce the cost of education for the parents and guardians thereby improving school attendance and reducing child labour. Government, non-governmental agencies, and interested policy actors should double their efforts and increase their focus and campaign against child labour to fast track the implementation of legislation against child labour.

## Supplementary Information

**Additional file 1.**

## Data Availability

The data sets used and/or analysed during the current study are available from the corresponding author on reasonable request.

## Abbreviations

AOR: Adjusted odds ratio
CRC: Conventions on the rights of the child
ILO: International Labour Organization
ICLS: International Conference of Labour Statisticians
JSS: Junior secondary school
LGA: Local government area
MICS: Multiple Indicator Cluster Survey
MSK: Musculoskeletal system
PCA: Principal Component Analysis
Q: Quartiles
SPSS: Statistical Package for Social Sciences
UBE: Universal Basic Education
UNICEF: United Nations Children’s Fund
UNTH: University of Nigeria Teaching Hospital
