# Supplementary material for: Prevalence and predictors of child labour among junior public secondary school students in Enugu, Nigeria: a cross-sectional study
Source: BMC Public Health. 2021 Jul 7;21:1339. doi: 10.1186/s12889-021-11429-w (PMC8262090; doi:10.1186/s12889-021-11429-w)
Supplement: Supplementary file 1 — Additional file 1. [file 12889_2021_11429_MOESM1_ESM.doc]

- - 1. **QUESTIONNAIRE**

**ASSESSMENT OF CHILD LABOUR AMONG PUBLIC SECONDARY SCHOOL STUDENTS IN ENUGU METROPOLIS.**

Dear respondent,

I am a Master of Public Health postgraduate student of Community Medicine Department, University of Nigeria Enugu Campus (UNEC). I am conducting a research on **Assessment of child labour among public secondary school students in Enugu metropolis**. The aim of the study is to ascertain the prevalence, perception, pattern, and factors associated with child labour among students in Enugu metropolis. This information is needed so that the public, government, and NGOs will become informed on this issue and will make laws/policies that will protect and promote the wellbeing of children. Please note that your identity and response will be kept confidential and strictly only for research purposes and your participation is voluntary. Thank you!

Instruction: Circle or fill as it applies. School -------------------------------------LGA -----------------------

| 1.Gender [1] Male [2] Female | 2.Age as at last birthday ------------- |
| --- | --- |
| 3.State of origin --------------- | 4.Tribe [1] Igbo [2] Hausa [3] Yoruba [4] Fulani [5] Others, specify-------- |
| 5.Class [1] JSS1 [2] JSS2 [3] JSS3 | 6.Religion [1] Christianity [2] Islamic [3] Traditional |
| 7.Birth order ----------- | 8.Total number of children in family--- |
| 9.Family size ----------------- | 10.Household type [1] Monogamous [2] Polygamous |
| 11.Family structure [1] Nuclear [2] Extended | 12.Custodian [1] Both parents [2] Single mother [3] Single father [4] Other relative [5] Unrelated guardian [6] Others, specify------ |
| 13.Highest education of father [1] Tertiary [2] Secondary [3] Primary [4] none [5] Others specify------ | 14.Highest education of mother[1] Tertiary [2] Secondary [3] Primary [4] none [5] Others specify------ |
| 15.Highest education of male custodian [1] Tertiary [2] Secondary [3] Primary [4] none [5] Not applicable | 16.Highest education of female custodian [1] Tertiary [2] Secondary [3] Primary [4] none [5] Not applicable |
| 17.Major source of income of father [1] Petty trading [2] Big business [3] Public servant [4] Employed in a private firm [5] Self-employed [6] Farmer [7] Unemployed [8] Others, specify-------- | 18.Major source of income of mother[1] Petty trading [2] Big business [3] Public servant [4] Employed in a private firm [5] Self-employed [6] Farmer [7] Unemployed [8] Others, specify------ |
| 19.Major source of income of male custodian[1] Petty trading [2] Big business [3] Public servant [4] Employed in a private firm [5] Self-employed [6] Farmer [7] Unemployed [8] Others, specify------[9]Not applicable | 20.Major source of income of female custodian[1] Petty trading [2] Big business [3] Public servant [4] Employed in a private firm [5] Self-employed [6] Farmer [7] Unemployed [8] Others, specify-------[9] Not applicable |
| 21.Primary care giver[1] Both parents [2] Single mother [3] Single father [4] Other relative [5] Unrelated guardian [6] Others, specify--- | 22.Type of house you live in [1] Duplex [2] 3-4 bedroom flat [3] 2 bedroom flat [4] Single room self-contained [5] Single rooms shard-amenities (yard) [6] Others specify----- |
| 23.House ownership [1] Yes [2] No | 24. Payment of rent [1] Yes [2] No [3] Others specify-------- |
|  | 25. Does your household own any of the following [1]Radio [2]Television [3]Air conditioner [4]Car [5] Fridge [6]Generator [7]Electric fan [8] Phone [9] Rechargeable light [10] Electric Iron |
| 25. Have you heard of child labour? [1] Yes [2] No | 26. What is your view about it? [1] Right [2] Wrong [3] None |
|  | **DID YOU DO ANY OF THE FOLLOWING ACTIVITIES (Q27-Q32) EVEN FOR ONLY ONE HOUR?** |
| 27. Do you think child labour should be encouraged in our society? [1] Yes [2] No | 28. Did you do any work or help on your own or household’s plot, farm, food garden or looked after animals? For example, growing farm produce, harvesting, or feeding, grazing or milking animals? [1] Yes [2] No |
| 29. Did you help in a family business or a relative’s business with or without pay, or run your own business? [1] Yes [2] No | 30. Did you produce or sell articles, handicrafts, clothes, food or agricultural products? [1] Yes [2] No |
| 31. Since past one week, did you engage in any other activity in return for income in cash or in kind, even for only one hour? [1] Yes [2] No | 32. Since past one week about how many hours did you engage in these activities, in total?--------------- |
| 33. Does the activity/Do these activities require carrying heavy loads? [1] Yes [2] No | 34. Does the activity/Do these activities require working with dangerous tools such as knives and similar or operating heavy machinery? [1] Yes [2] No |
| 35**.ABOUT YOUR WORK ENVIRONMENT**, Circle the ones that apply [1] Exposed to dust, fumes or gas [2] exposed to extreme cold, heat or humidity [3] Loud noise or vibration [4] Required to work at heights [5] Required to work with chemicals such as pesticides, glues, explosives etc [6] Exposed to other things, processes or conditions bad for your health or safety | 36. Since past one week, did you fetch water for household use? [1] Yes [2] No |
| 37. In total, how many hours did you spend on fetching water for household use, since past one week?--------- | 38. Since past one week, did you collect fire wood for household use? [1] Yes [2] No |
| 39. In total, how many hours did you spend on collecting firewood for household use, since past one week?------ | 40. Since past one week, circle the ones you did for your household [1] Shopping for the household [2] Cooking [3] Washing dishes or cleaning around the house [4] Washing clots [5] caring for children [6] Caring for someone old or sick [7] Other household tasks |
| 41. Since past one week, about how many hours did you engage in the above activities, in total----------------- | 42. Type of activities you are usually involved in [1]Street hawking [2]Selling in kiosks [3]Car wash [4] Domestic work [5] Barrow pushing [7] Farm work [8] apprenticeship [9] Others, specify--------- |
| 43. Reason for being involved in these activities[1] Assist parents financially [2] Gain experience [3] Parents / Guardian forces me [4] Prepares for future work [5] Help in family business [6] Others, specify------ | 44. Challenges ever experienced[1] Minor injuries [2] Major injuries [3] Physical abuse [4] Sexual abuse [5] Road accident [6] School drop-out [7] Frequent lateness to school [8] Poor academic performance [9] Illness due to cold [10] My peers laughed at me [11] Robbed by hoodlums [12] Others specify--- |
| 45. Age when started to carry out these activities------------- | 46. Number of working children in the household…….. |
| 47. Self-ownership of business[1] Yes [2] No  If yes, please specify type------ | 48. your weekly income---------------- |
| 49. Who controls your earnings? [1] Parents [2] Siblings [3] Self [4] Custodian [5] Keep and spend on my own [6] Others, specify----- | 50. Are you satisfied with the work you do? [1] Yes [2] No |
| 51. what measures do you think can be applied to control child labour in our society [1] Free education [2] Control of household poverty [3] Public enlightenment [4] Enforcement of child laws |  |
